# Polyorchidism: two case reports and a review of the literature

**DOI:** 10.1186/1752-1947-8-464

**Published:** 2014-12-25

**Authors:** Suheil Artul, George Habib

**Affiliations:** 23 Radiology Department, EMMS Hospital Nazareth, Bar Ilan University, Faculty of Medicine, P. O. Box 11, 16100 Nazareth, Israel; 24 Rheumatology Clinic, Nazareth Hospital, P. O. Box 11, 16100 Nazareth, Israel

**Keywords:** Polyorchidism, Scrotum, Testes, Ultrasound

## Abstract

**Introduction:**

Polyorchidism is a very rare anomaly that is defined by the presence of more than two testes. Although its presentation is primarily as triorchidism, cases of four testes have also been reported in the literature.

**Case presentation:**

In this report, we describe color Doppler ultrasound findings in two cases. Patient 1 was a 37-year-old Arabic man with a scrotal mass and a double testicle in the right hemiscrotum visualized by ultrasound. Patient 2 was an 11-year-old Arabic boy with an inguinal mass resulted to be an additional testicle in the inguinal canal. The echogenic texture and vascular flow of supernumerary testicles in question were similar to those of the normal testicles; however, their size was smaller. After 3 years of follow-up, the tertiary testes in the two patients remained stable in both size and echogenicity.

**Conclusion:**

Ultrasound plays a crucial role in the evaluation of masses. Notably, inguinal or scrotal masses should not always be considered as lymph nodes or tumors. Indeed, a radiologist should always keep polyorchidism in mind when such masses are encountered.

## Introduction

Polyorchidism or supernumerary testicles are defined by the presence of more than two testicles. It is a very rare anomaly. Only about 200 cases have been reported in the literature so far. In this report, we report the color Doppler ultrasound findings in two patients with polyorchidism that presented as asymptomatic masses, and we review the pertinent literature.

## Case presentations

### Patient 1

**Figure 1:**
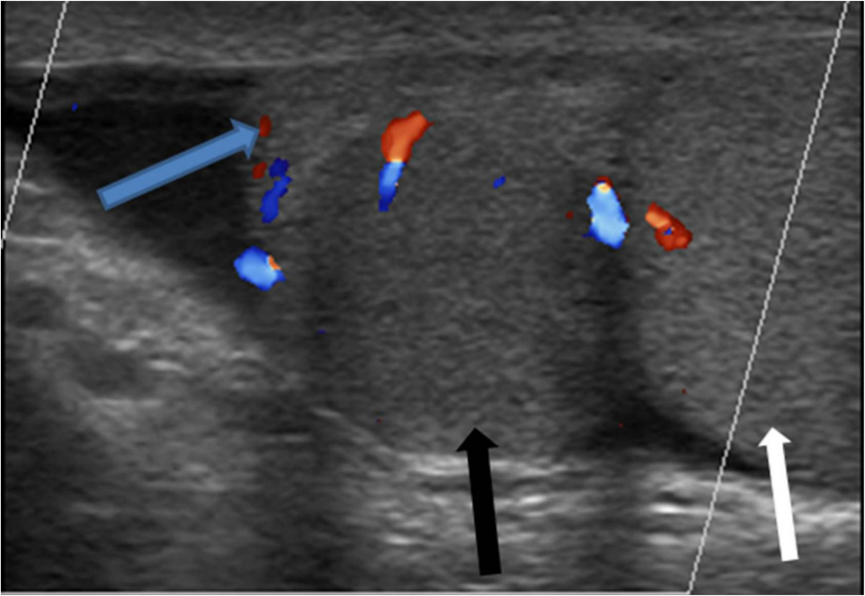
**Ultrasound of patient 1.** Longitudinal section of a color Doppler ultrasound shows normal testis (white arrow) in the right hemiscrotum and a 1.5cm oval mass in the right hemiscrotum (black arrow), adjacent to the upper pole of the right testis and slightly compressing the epididymis (blue arrow). This mass has the same echogenicity and normal flow as the testis nearby.

A 37-year-old Arabic man was referred by his family physician to our ultrasound unit for evaluation of a palpable, painless mass in the right scrotum that he had had for at least 3 years at least. Color Doppler ultrasound (Figure 1) showed a normal testicle on each side and a 1.5cm oval mass in the right hemiscrotum adjacent to the upper pole of the right testicle, slightly compressing the epididymis. Additionally, the mass had the same echogenicity and normal flow signs as the nearby testicles.

It was therefore presumed to be a third testicle, and the patient was managed with follow-up only, initially every 3 months and then after one year every 6 months. The sonographic findings were stable during this follow-up period.

### Patient 2

**Figure 2:**
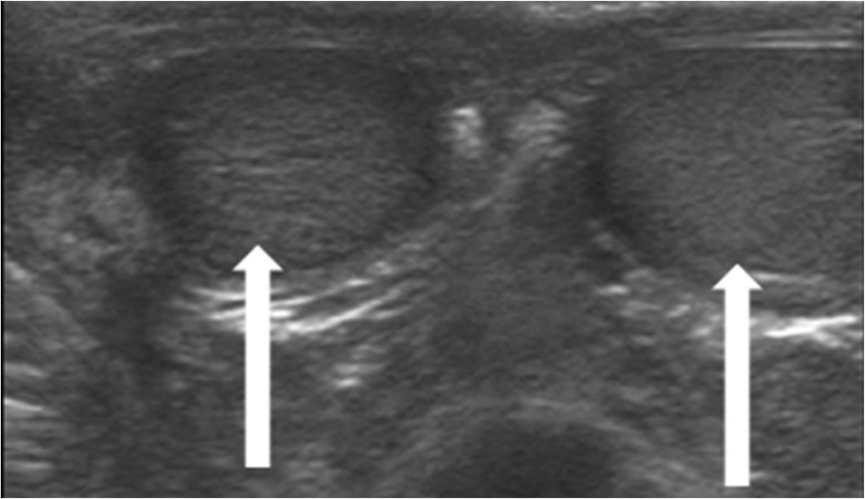
**Grayscale ultrasound of patient 2.** Image shows a normal testis on each side of the scrotum (white arrows).

An 11-year-old Arabic boy was referred electively to our ultrasound unit for evaluation of a painless mass in the right inguinal area that was incidentally detected by his father while they were bathing. A grayscale ultrasound showed a normal testicle on each side of the scrotum (Figure 2).

**Figure 3:**
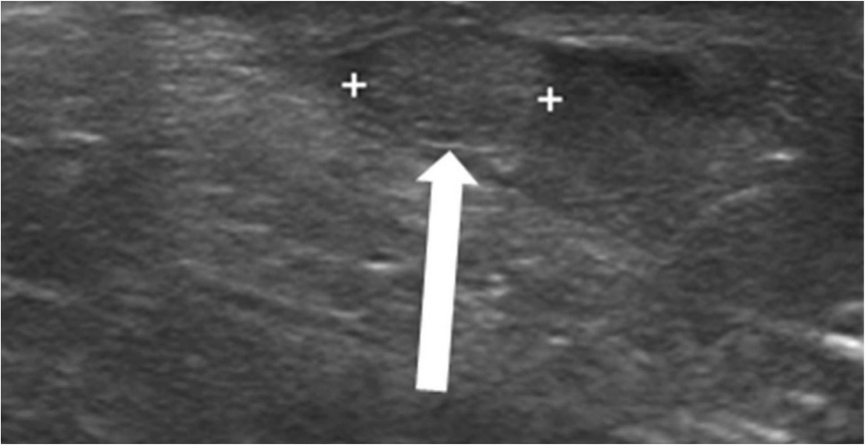
**Ultrasound of the right inguinal canal in patient 2.** Image shows a 0.7cm oval mass that resembles a testis in its echotexture (white arrow).

An ultrasound of the boy’s right inguinal canal showed a 0.7cm oval mass resembling a normal testicle in its echotexture (Figure 3). It was easily pushed down manually into the scrotum.

**Figure 4:**
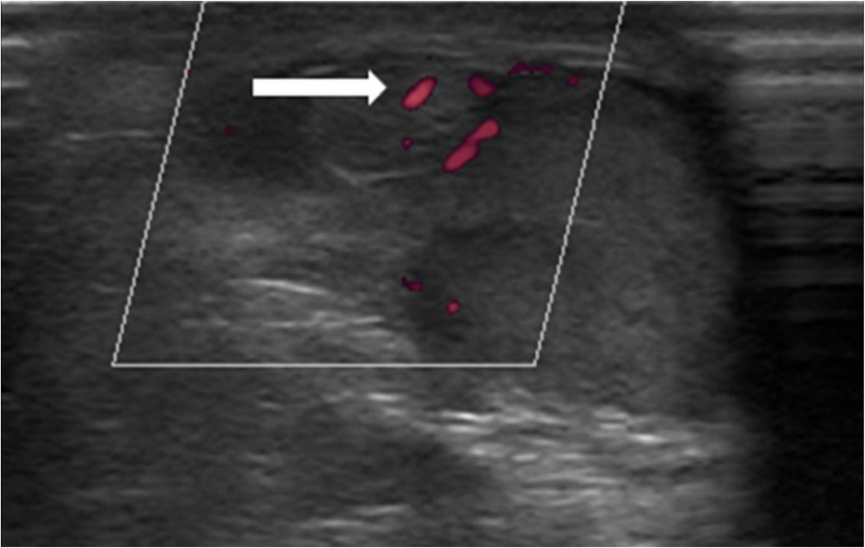
**Power Doppler ultrasound of the mass in patient 2.** Image shows normal flow (white arrow).

A power Doppler ultrasound showed a normal flow in this mass (Figure 4).

This mass was diagnosed as a retractile third testicle, and no change in size or echogenicity has been observed during more than 3 years of follow-up.

## Discussion

The first histological description of polyorchidism was published in 1880 by Ahlfeld. The first clinical case was reported by Lane in 1895 [1]. Polyorchidism is a very rare anomaly, which is defined by the presence of more than two testicles. Triorchidism—three testicles—is the most common form of polyorchidism, in which an extra testicle is usually found on the left side. The exact etiology of polyorchidism is still unknown. However, it could be related to an embryological developmental abnormality during the formation of the testicles [2]. Usually, patients present with an asymptomatic mass. However, they are significantly prone to developing a testicular torsion and malignancy.

The color Doppler ultrasound appearance of polyorchidism is a scrotal oval mass with a flow similar to that of the normal testicle. Spermatogenesis in the supernumerary testicle is normal in about 50% of cases. This fact explains the persistent fertility of these patients after bilateral vasectomy. Indeed, when bilateral vasectomy is performed, fertility remains normal.

Most patients with polyorchidism have a normal 46,XY karyotype. However, chromosomal abnormalities such as a 46,XX karyotype with XY mosaicism and deletion of the long arm of chromosome 21 have been reported. In polyorchidism, secondary sexual characteristics are the same as in typical individuals of similar age [2, 3].

Leung [3] and Singer and colleagues [4] classified embryonic polyorchidism into four types. Type A: The supernumerary testicle lacks an epididymis and vas deferens, as in our patient 2. Type B: The supernumerary testicle shares an epididymis and vas deferens with another testicle, as in our patient 1. Type C: The supernumerary testicle has its own epididymis and shares the vas deferens with a regular testicle. Type D: The supernumerary testicle has complete duplication of testicles, epididymides and vas deferens.

Polyorchidism is usually diagnosed on the basis of color Doppler ultrasound, further supported by magnetic resonance imaging (MRI) [5]. In both of our patients, MRI was recommended; however, both patients preferred to be followed up with ultrasound. This was due to claustrophobia in patient 1 and for technical reasons in patient 2. The supernumerary testicle generally demonstrates echotexture and vascular flow similar to those of the normal testicles. MRI can be helpful if an ultrasound-based diagnosis is not definitive [6]. The supernumerary testicle has the same MRI features as a normal testicle: intermediate signal intensity on T1-weighted images and high signal intensity on T2-weighted images [7]. The differential diagnosis of polyorchidism includes an extra-testicular or para-testicular mass, such as hydroceles, varicoceles, lipomas, tumors and/or hernia.

The management of polyorchidism should usually be resection of the dysplastic testicle without a duct; otherwise, the supernumerary testis should be retained and followed up by ultrasound yearly because of its contribution to spermatogenesis. However, any suspected malignancy of the supernumerary testicle should be managed by orchiectomy. If malignancy is not suspected, the patient can be followed up conservatively, as we did in our two patients.

In one study, Bergholz and Wenke reviewed 140 histologically confirmed cases of polyorchidism [8]. Triorchidism was the most common type. Quadriocrchidism was observed in six cases (4.3%). Most supernumerary testicles (64%) were drained by a vas deferens. The median patient age at diagnosis was 17 years. The left side demonstrated predominance, being affected in 64.5% of the patients. Most cases were detected during surgery performed to treat other symptoms, including inguinal hernia, undescended testicle, testicular torsion or scrotal pain. Only 16% of patients complained of a palpated mass without any symptoms. Tumors were found in nine cases (6.4%), of which eight were malignant and one was benign. Most of these malignancies were found in undescended supernumerary testicles.

The most common location of the supernumerary testis is within the scrotum. However, it may be present with a maldescended testis, or it may itself be maldescended in patients with two testicles in the scrotum.

Anomalies associated with polyorchidism generally include inguinal hernia in cryptorchidism, testicular torsion and microlithiasis [1].

Polyorchidism in general is an incidental finding associated with hydrocele, epididymitis, varicocele, infertility, retractile testes and hypospadias. Reports in the literature also have described an increased risk of testicular malignancy (6.4%) in the presence of polyorchidism [5]. Malignant transformation may occur regardless of the location of the supernumerary testicle. Most commonly reported neoplasms are embryonal carcinomas, germ cell tumors and seminomas. Extratesticular rhabdomyosarcoma and adenoma of rete testis in a supernumerary testicle have also been noted [7].

Leung [3] recommended testicular biopsy for diagnosis; however, we believe that contemporary ultrasound technology allows diagnosis of this condition with a degree of high certainty. Chung and Yao [9] reported that high-resolution sonography is an effective and noninvasive method for accurate diagnosis of polyorchidism without the need for exploratory surgery. Yalçınkaya and colleagues [5] reported that, in most cases, sonography alone is diagnostic. MRI accordingly may provide additional information in complicated cases of polyorchidism. Savas and colleagues concluded that it appears to be safe to preserve a viable intrascrotal supernumerary testicle found incidentally during surgery, provided that the patient is followed up in the long term [10].

Another condition that mimics polyorchidism is splenogonadal fusion, which is essentially a very rare congenital anomaly. In this condition, the spleen, gonad, epididymis and vas deferens are fused. Sonography reveals a mass with a testicle of similar echogenicity, and it may mimic the appearance of polyorchidism. Hence, when a splenogonadal fusion is suspected, a technetium sulfur colloid scan should be performed to confirm the presence of ectopic splenic tissue [11].

## Conclusion

Polyorchidism is a very rare anomaly. Doppler ultrasound is the preferred method for evaluation, diagnosis and follow-up of this condition, and it can help avoid the need for surgery. Radiologists and urologists must be aware of this condition when evaluating any scrotal mass. These patients should be followed up yearly by ultrasound for life, but young patients with a third testicle that does not contribute to spermatogenesis, as in our 11-year-old patient, should undergo resection. MRI is rarely needed nowadays. Furthermore, excision is recommended when any change in echogenicity, shape and focal masses occur in the supernumerary testicle.

## Consent

Written informed consent was obtained from patient 1 and the legal guardian of patient 2 for publication of this case report and accompanying images. A copy of the written consent is available for review by the Editor-in-Chief of this journal.

## Abbreviations

MRI: Magnetic resonance imaging.
